# In vivo microstructural analysis of the humeral greater tuberosity in patients with rotator cuff tears using multidetector row computed tomography

**DOI:** 10.1186/1471-2474-15-351

**Published:** 2014-10-21

**Authors:** Yoshihiro Sakamoto, Akira Kido, Kazuya Inoue, Goro Sakurai, Tomohisa Hashiuchi, Mitsuru Munemoto, Yasuhito Tanaka

**Affiliations:** Department of Orthopaedic Surgery, Nara Medical University, 840 Shijocho, Kashihara, Nara 634-8521 Japan; Department of Orthopaedic Surgery, Nishinara Chuo Hospital, Nara, Japan

**Keywords:** Osteoporosis, Microstructure, Trabecular structure, Rotator cuff tear, Suture anchor pullout

## Abstract

**Background:**

In arthroscopic surgery, the suture anchor technique has become popular for rotator cuff repair. Preoperative evaluation of the bone microstructure is of utmost importance because, especially in elderly patients, osteoporotic changes may cause anchor pullout, which results in failure of rotator cuff repair. Many groups have reported humeral microstructural analysis; however, most studies were experiments using porcine specimens or human cadavers. In this study, we used multidetector row computed tomography to successfully perform in vivo evaluation of the bone microstructure of the humeral greater tuberosity in patients with rotator cuff tears.

**Methods:**

Ten patients were examined. Regions of interest were defined in six quadrants of the greater tuberosity (medial, lateral, and far lateral rows of the anterior and posterior areas). The local bone mineral density and the trabecular microstructural parameters, including the mean bone volume to total volume (BV/TV), trabecular thickness, trabecular separation, and structure model index (SMI), were measured using bone analysis software.

**Results:**

The BV/TV of the posteromedial region was highest and the SMI of the posteromedial region was lowest. These findings suggest that the bone quality of the posteromedial portion is the highest within the greater tuberosity.

**Conclusion:**

Because the bone quality may be correlated with the pullout strength of suture anchors, our method can help to understand the individual and regional variance in bone quality and may lead to the creation of personalized surgical protocols.

**Electronic supplementary material:**

The online version of this article (doi:10.1186/1471-2474-15-351) contains supplementary material, which is available to authorized users.

## Background

Rotator cuff tears affect the overall quality of life of individual patients and rotator cuff repair has an important role in minimizing the societal burden of rotator cuff disease [1]. Furthermore, because of increases in physical and sport activities in older populations, rotator cuff repair has been playing an increasingly important role in these individuals [2, 3]. There are two main rotator cuff repair methods: the suture anchor technique and the bone tunnel technique [4]. With advances in arthroscopic surgery, the suture anchor technique has become more popular because of its ease and speed [5–7]. However, because of osteoporotic bone changes in older individuals, this technique is often associated with anchor pullout before tendon healing occurs, which results in tendon rupture and failure of rotator cuff repair at the rate of 10% [8–11]. Thus, preoperative evaluation of bone quality is considered to be very important.

Many groups have reported that the bone quality of the humerus is correlated with the pullout strength of suture anchors [8, 12–14]. Barber et al. [8] and Tingart et al. [14] described the correlation between bone mineral density (BMD) and pullout strength in the greater tuberosity using dual-energy X-ray absorptiometry or quantitative computed tomography. However BMD measurements alone have limitations because there is evidence that only a small fraction of the reduction in fracture with therapy can be accounted for by the increase in BMD [15]. In fact, factors other than BMD, such as bone structure and turnover rate of bone remodeling, contribute to bone fragility [16]. Ito et al. [17] described the correlations between microstructure parameters and bone strength. Poukalova et al. and Yakacki et al. [12, 18] investigated the relationships between microstructure and suture anchor pullout strength. Kirchhoff et al. [19] analyzed microstructure of the osteoporotic humeral head. However, the applicability of these findings are limited, because all of these studies were performed only using porcine specimens or human cadavers.

Here, we hypothesized that bone microstructure of the humerus could be measured in vivo using multidetector row computed tomography (MDCT), because MDCT is a new technique that has a substantially higher spatial resolution than standard spiral CT and thus promises to improve the assessment of trabecular bone microstructure [17]. Therefore, the aim of this study was to evaluate microstructure of the greater tuberosity of the humeral head in patients with rotator cuff tears and to explore individual and regional variance of bone quality in vivo. To our knowledge, this is the first report on in vivo analysis of the microstructure of the humeral head in patients with rotator cuff tears.

## Methods

This research was performed following the Declaration of Helsinki principles. The study was approved by our institution’s ethics committee: Nara Medical University Ethics Committee (reference number 656). Written informed consent for participation in this study and the publication of their individual clinical details was obtained from each participant or, where participants are children, a parent or guardian. All patients consented to participation and the publication.

### Patients

A continuous series of 10 patients from our hospital were included in this study. All patients were diagnosed with a rotator cuff tear and referred to our department for the surgical treatment. Clinical evaluation, X-ray, computed tomography (CT) and magnetic resonance imaging (MRI) were performed for the purpose of preoperative planning. Arthroscopic rotator cuff repairs were performed from January through April 2012. The patients comprised seven men and three women with a median age of 59.9 years (range, 50–72 years) (Table 1). According to the DeOrio and Cofield classification [20], there were nine small and one medium rotator cuff tears. Patients who met the following criteria were excluded: previous operation, fracture or infection of the affected shoulder, moderate to severe glenohumeral osteoarthritis, or cuff tear arthropathy with a large or massive rotator cuff tear.

**Table 1:** **The details of the patients**

Case	Age	Sex	Tear muscle	Size
1	58	Female	Supraspinatus	Small
2	69	Male	Supraspinatus	Small
3	51	Male	Supraspinatus	Small
4	58	Male	Supraspinatus	Small
5	56	Female	Supraspinatus	Small
6	65	Male	Supraspinatus	Small
7	50	Male	Supraspinatus	Small
8	53	Male	Supraspinatus	Small
9	67	Female	Supraspinatus	Small
10	72	Male	Supraspinatus	Medium

### Imaging conditions

Before surgery, an MDCT scan was performed with a Brilliance CT 64-channel scanner (Philips, Amsterdam, Netherlands) using a standardized protocol (120 kV, 248 mA, collimation of 0.67 mm, and reconstruction index of 0.3 mm) for the bone quality evaluation. The scans were performed under the following conditions: field of view of 200 mm and pixel matrix of 512 × 512.

### Regions of interest

**Figure 1:**
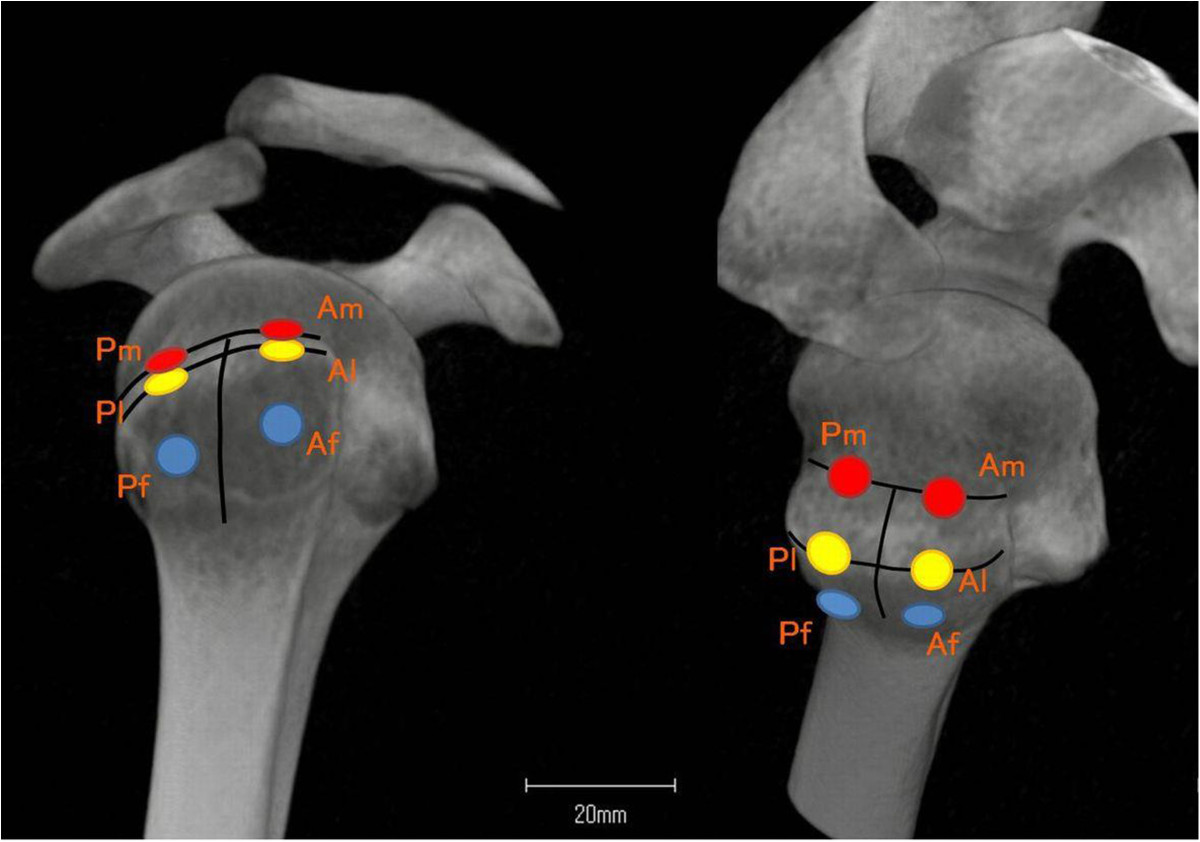
**Six regions of interest (ROIs) were defined within the greater tuberosity of the humeral head.**

**Figure 2:**
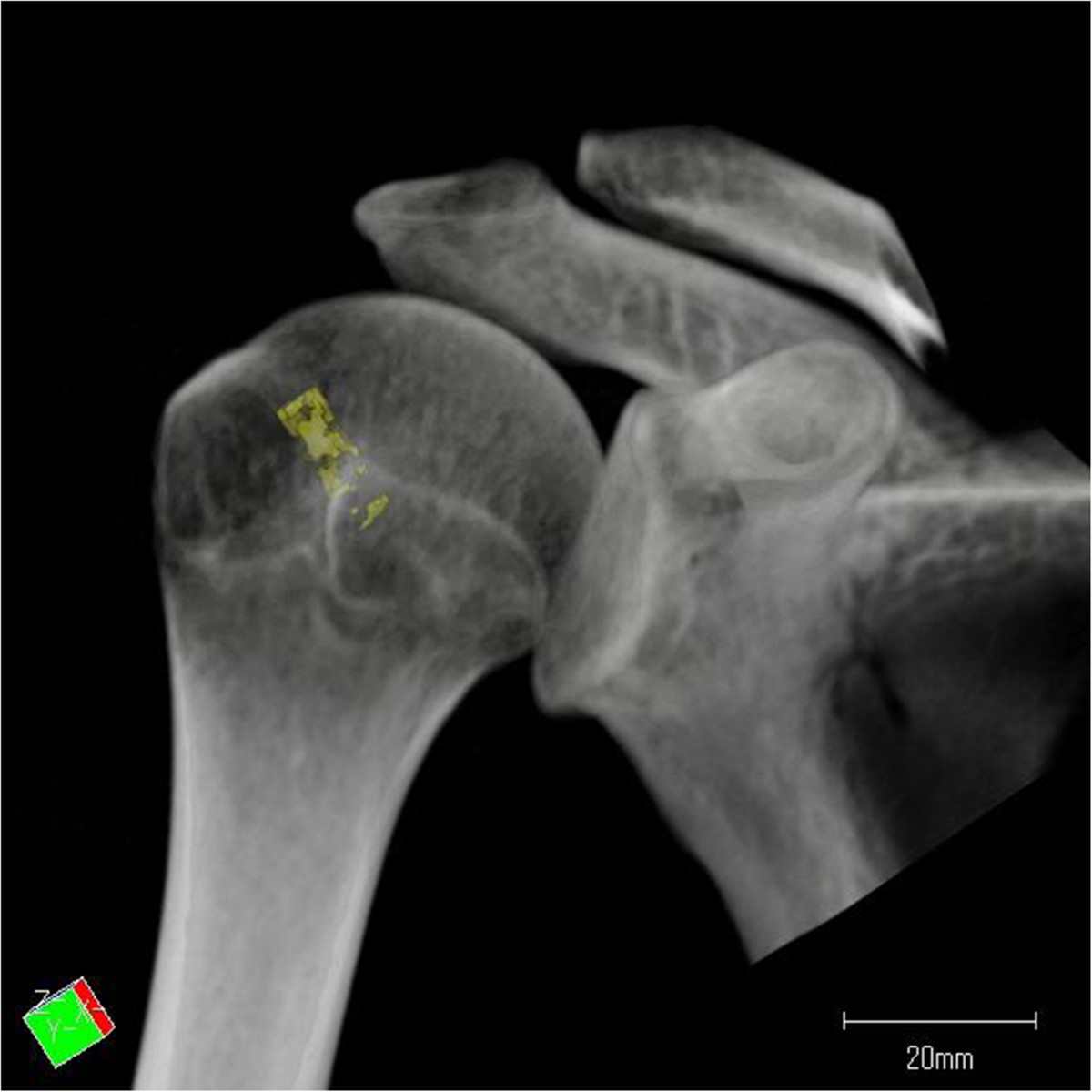
**Each ROI was placed at a 45° angle to the greater tuberosity and 5 mm under the surface of the cortical bone.**

To perform the morphometric analysis, specific regions of interest (ROIs) were defined within the greater tuberosity of the humeral head (Figure 1). These ROIs were designed with the suture anchor positioning in arthroscopic rotator cuff repair. The borders of the footprint were defined in each case. Next, the greater tuberosity was divided into two equally sized areas (area A and area P). Area A was set on the anterior side of the greater tuberosity, and area P was set on the posterior side. Three rows were defined within each area: one medial row directly adjacent to the articular surface (Am, Pm), one lateral row along the lateral edge of the footprint (Al, Pl), and one far lateral row 1 cm from the lateral edge of the footprint (Af, Pf). Each ROI had a cylindrical shape with a diameter of 5 mm and a depth of 15 mm, corresponding to the average volume of currently used suture anchors. Each ROI was placed at a 45° angle to the greater tuberosity [21]. The ROIs were set 5 mm under the surface of the cortical bone to omit cortical bone artifact (Figure 2).

### Structure analysis

**Figure 3:**
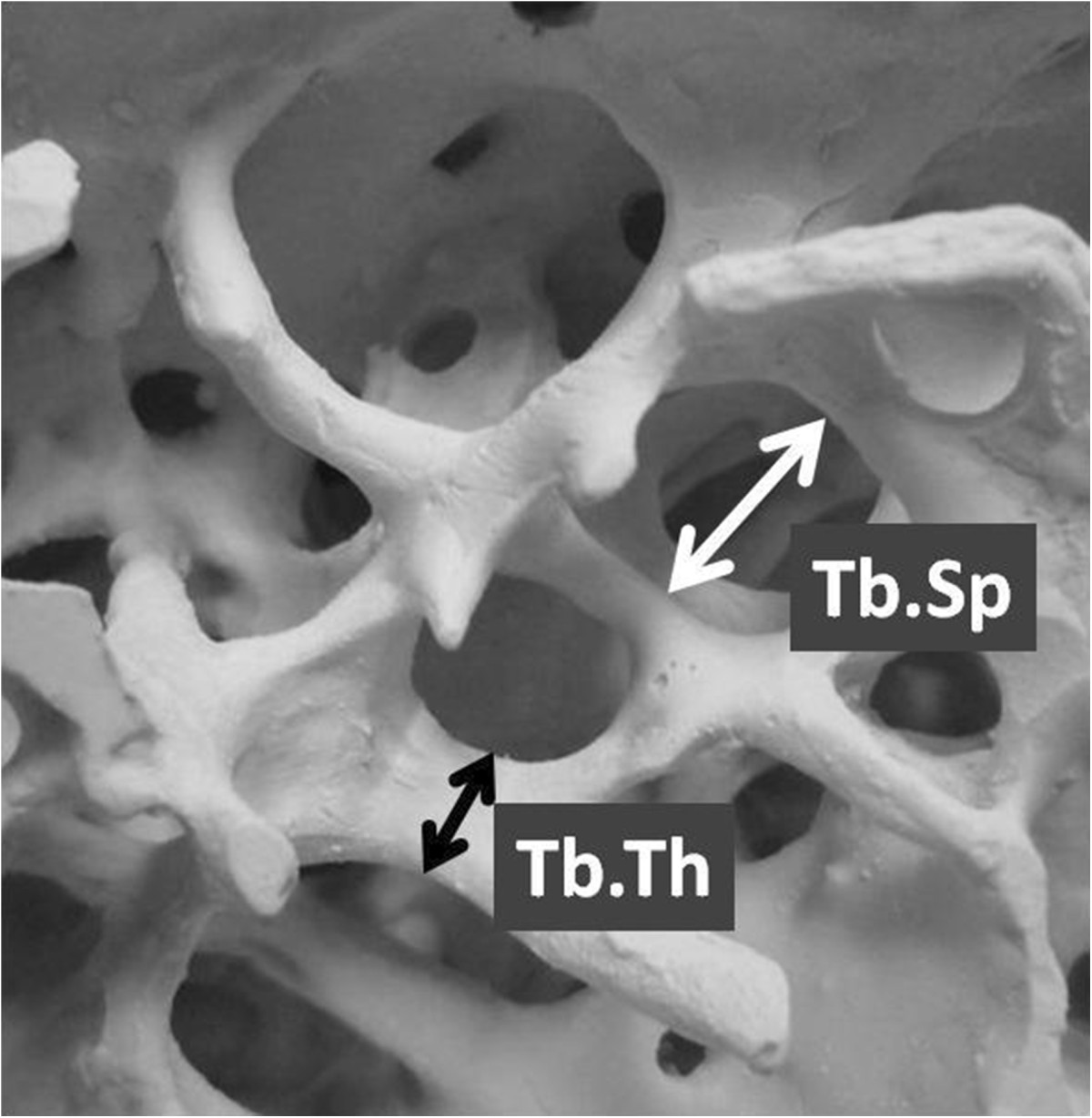
**Trabecular microstructural parameters were measured using three-dimensional (3D) image analysis software.** The black arrow indicates the trabecular thickness (Tb.Th, μm), and the white arrow indicates the trabecular separation (Tb.Sp, μm).

After MDCT image data were transferred to a workstation, BMD (mg/cm^3^) and the trabecular microstructure parameters were measured using a three-dimensional (3D) image analysis software (TRI/3D-BON; RATOC System Engineering Co., Tokyo, Japan). To establish the interobserver reliability for measuring each parameter, two experienced shoulder surgeons (Y.S. and K.I.) input all ROIs manually under three-dimensional coordinates on this software, and then each parameter were measured automatically according to the software program. After 30 minutes of the instruction, input was carried out independently. To demonstrate the intraobserver reliability of the measurements, 2 examination sessions were carried out at an interval of 3 weeks by Y.S. Grayscale images were segmented using a median filter to remove noise with a fixed threshold to extract mineralized bone components. We used a discriminant analysis method of image thresholding based on the density histogram of a selected ROI to ensure consistent image thresholding across all subjects studied. Isolated small particles in the marrow space and isolated small holes in bone were removed using a cluster-labeling algorithm in order to remove the small noise in the binary extraction. The measurement parameters calculated in 3D were the bone volume fraction that indicates bone volume/total volume (BV/TV,%), trabecular thickness (Tb.Th, μm), trabecular separation (Tb.Sp, μm), and structure model index (SMI) (Figure 3). The SMI is used to evaluate whether trabecular bone is rod-like or plate-like; a smaller value indicates a more plate-like structure [22, 23]. It was established that good bone quality included the higher BMD, higher BV/TV, higher trabecular thickness, lower trabecular separation and the lower SMI [12, 17, 18].

### Statistical analysis

Statistical analyses were performed using the statistical software R (Ver. 3.0.1). All data were divided by the average for each individual because we used the optimal threshold value for each individual The data are presented as mean ± standard deviation (mean ± SD). The trabecular microstructure parameters and BMD among all ROIs were statistically evaluated by analysis of variance (ANOVA) and Tukey’s test. Interclass and Intraclass correlation coefficients were used to assess interobserver and intraobserver reliability. Statistical significance was established at P <0.05.

## Results

### BMD

**Figure 4:**
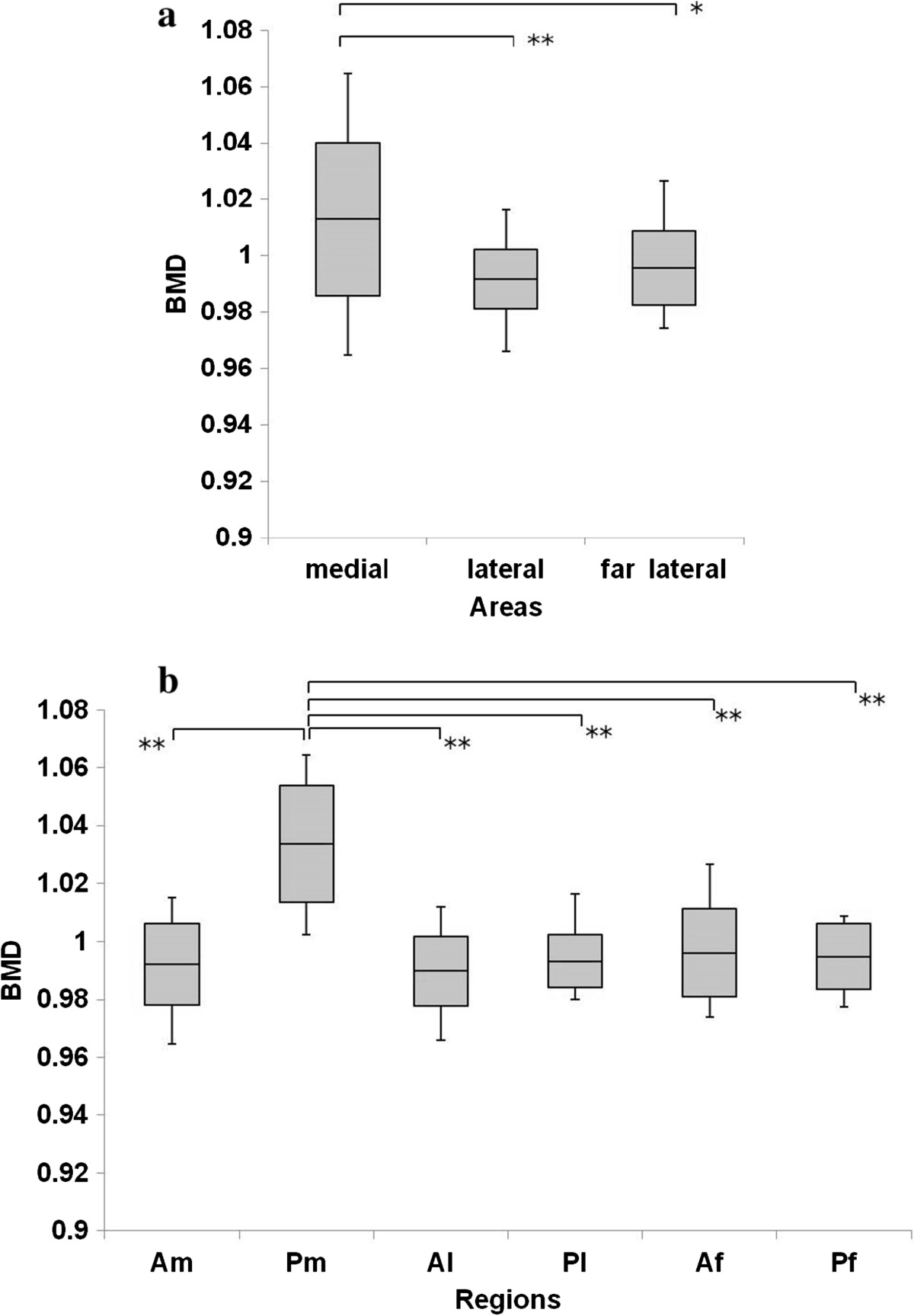
**Bone mineral density (BMD) in the greater tuberosity. (a)** BMD in the medial area was significantly higher than that in the lateral and far lateral areas. **(b)** BMD in Pm was significantly highest among all ROIs. *P <0.05, **P <0.01.

The BMD of the medial row (Am, Pm) was significantly higher than that of the lateral row (P = 0.002) and far lateral row (P = 0.012) (Figure 4a). The BMD in Pm was significantly highest among all ROIs (Am, P <0.001; Al, P <0.001; Pl, P <0.001; Af, P <0.001; Pf, P <0.001) (Figure 4b). The BMD was significantly correlated with the BV/TV (R = 0.77). The intraobserver reliability was good, with values of 0.78. The interobserver reliability was also good at 0.74.

### Microstructural parameters

**Figure 5:**
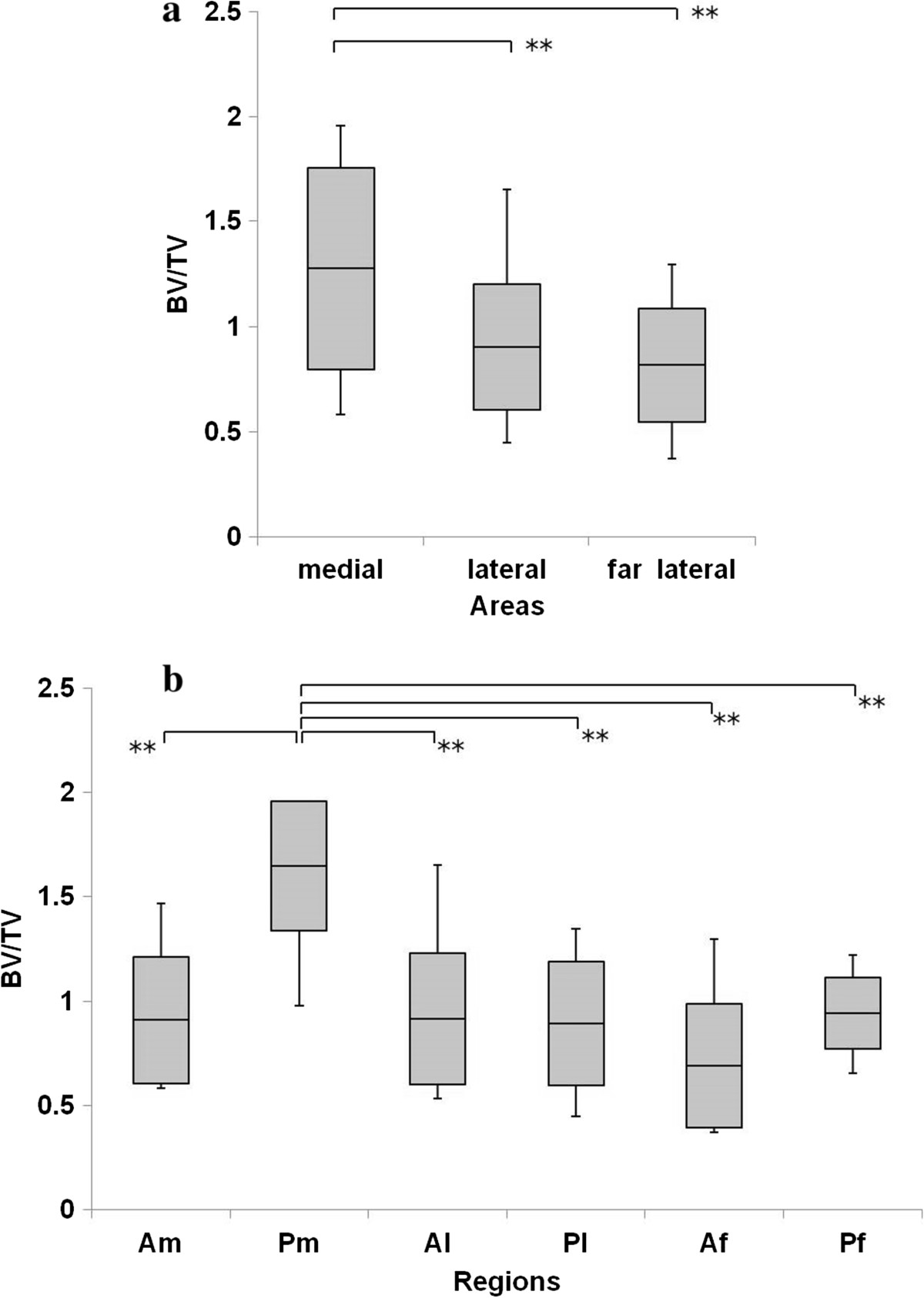
**BV/TV in the greater tuberosity. (a)** BV/TV in the medial area was significantly higher than that in the lateral and far lateral areas. **(b)** BV/TV in Pm was the highest among all ROIs. *P <0.05, **P <0.01.

**Figure 6:**
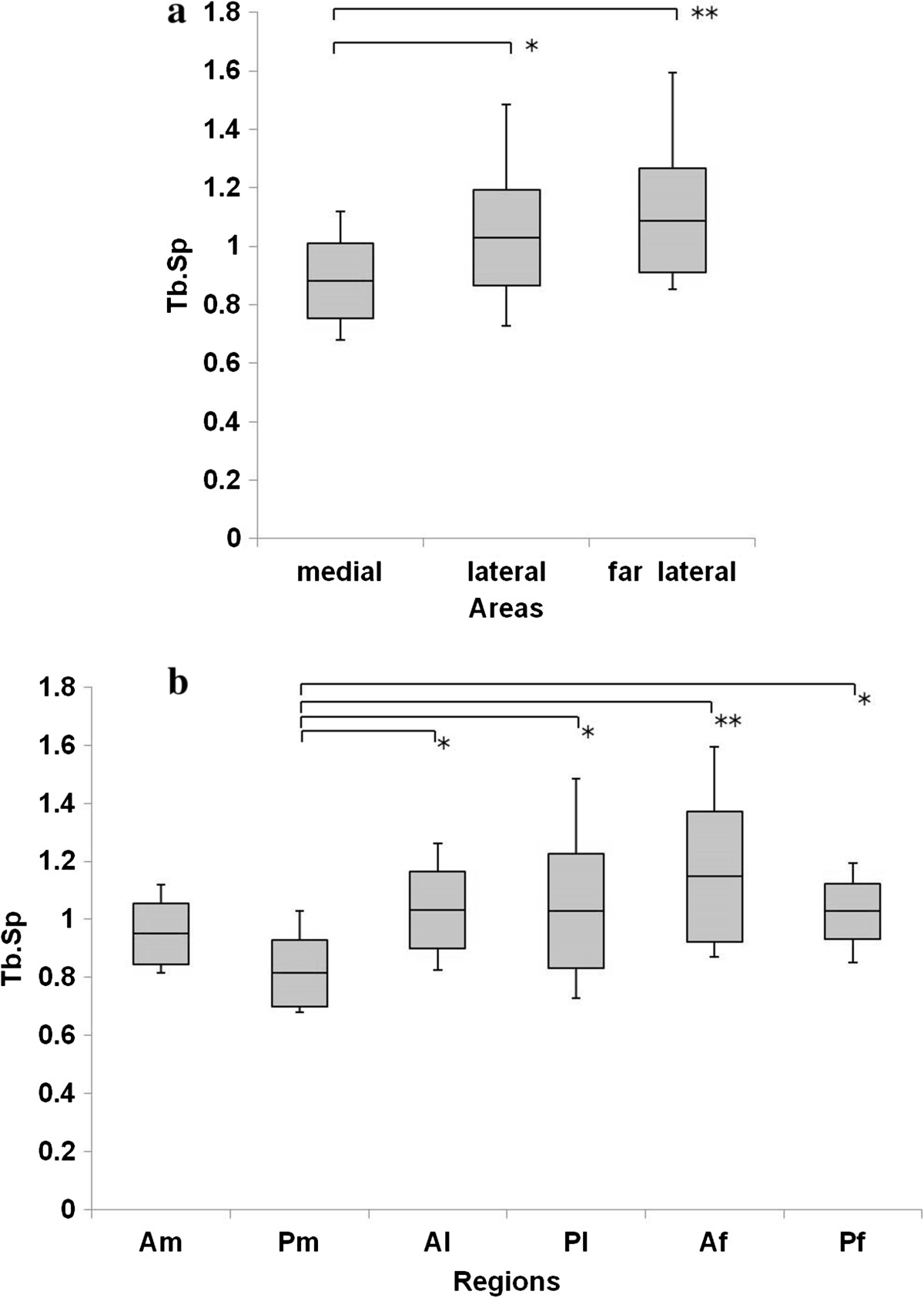
**Trabecular separation (Tb.Sp) in the greater tuberosity. (a)** Tb.Sp in the medial area was significantly lower than that in the lateral and far lateral areas. **(b)** Tb.Sp in Pm was significantly lower than that in Al, Pl, Pf, and Af. *P <0.05, **P <0.01.

**Figure 7:**
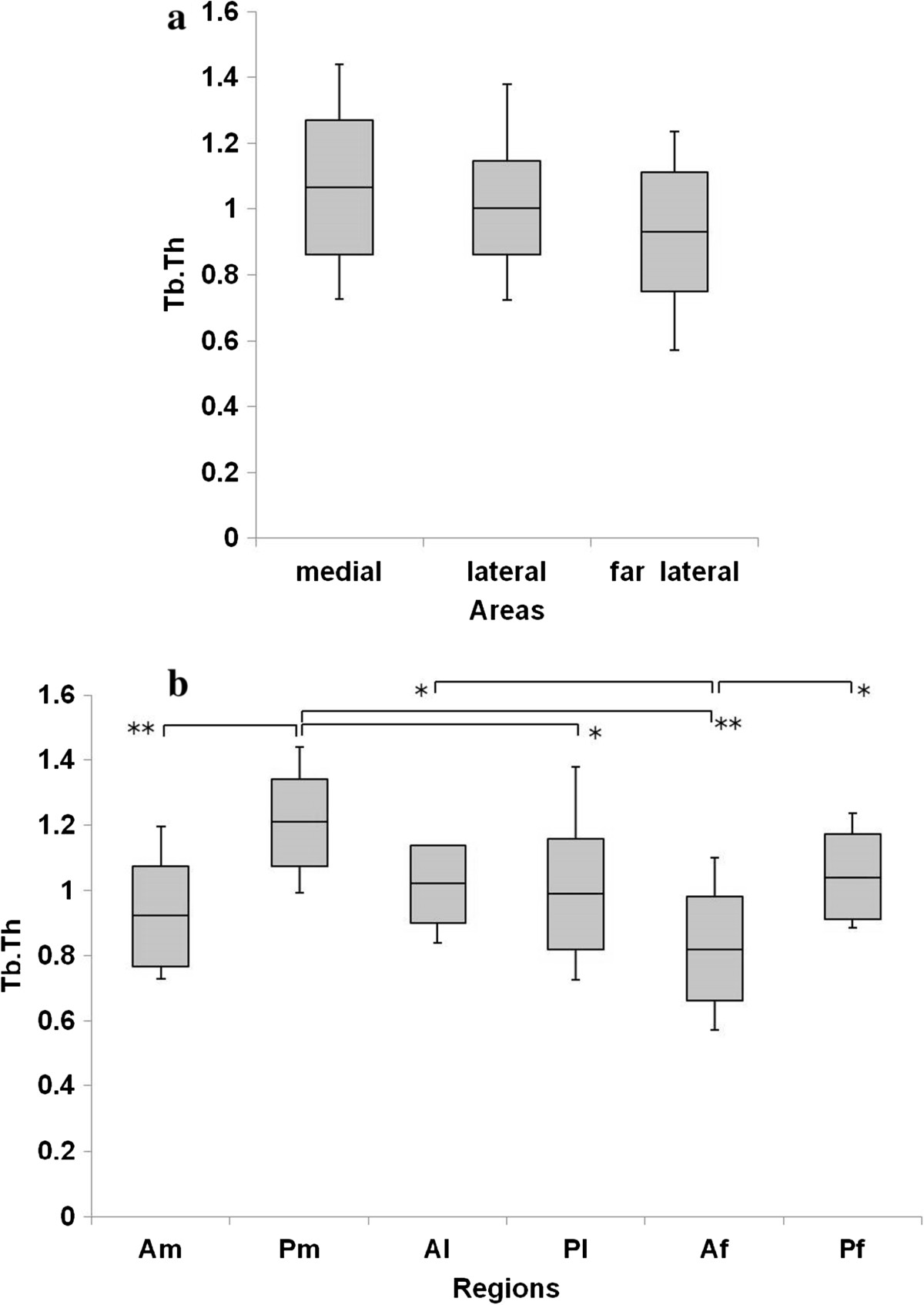
**Trabecular thickness (Tb.Th) in the greater tuberosity. (a)** There were no significant differences in the Tb.Th. **(b)** Tb.Th in Pm was significantly higher than that in Pl, Am, and Af. The Tb.Th in Af was significantly lower than that in Al and Pf. *P <0.05, **P <0.01.

**Figure 8:**
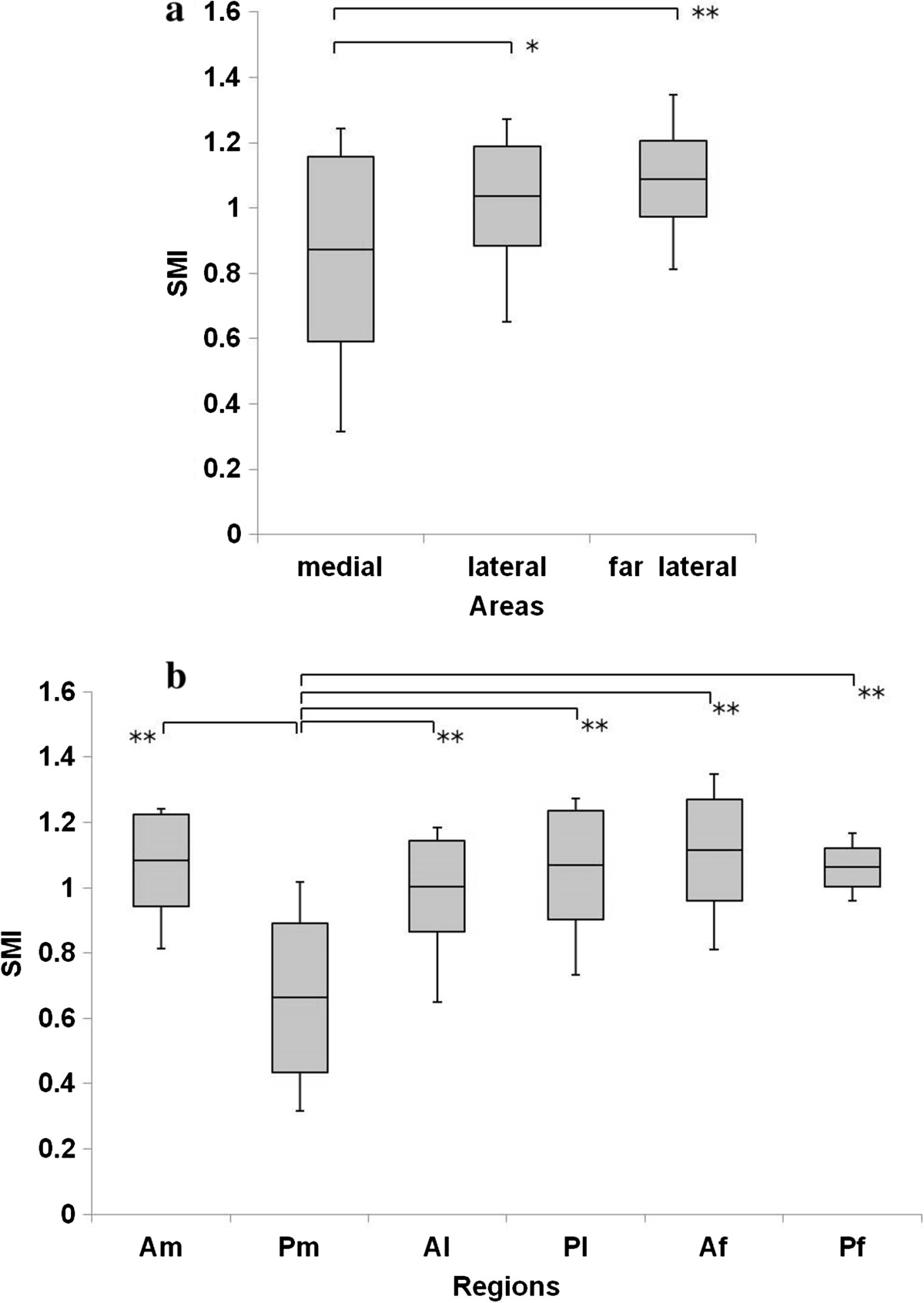
**Structure model index (SMI) in the greater tuberosity. (a)** SMI in the medial area was significantly lower than that in the lateral and far lateral areas. **(b)** SMI in Pm was significantly lowest among all ROIs. *P <0.05, **P <0.01.

Figure 5a shows that the BV/TV in the medial row was significantly higher than that in the lateral (P = 0.005) and far lateral rows (P = 0.001). The BV/TV in Pm was the highest among all ROIs (Am, P <0.001; Al, P <0.001; Pl, P <0.001; Af, P <0.001; Pf, P <0.001). The trabecular separation in Pm was significantly lower than that in Al, Pl, Af, and Pf (Al, P = 0.028; Pl, P = 0.031; Af, P <0.001; Pf, P = 0.031) (Figure 6a, b), and the trabecular thickness in Pm was significantly higher than that in Am, Pl, and Af (Am, P = 0.001; Pl, P = 0.015; Af, P <0.001) (Figure 7a, b). The SMI in Pm was significantly lowest among all ROIs (Am, P <0.001; Al, P <0.001; Pl, P <0.001; Af, P <0.001; Pf, P <0.001) (Figure 8a, b).

Overall, these results suggest that the posterior medial portion possesses the most ideal plate-like structure with the highest bone quality, while the lateral row and far lateral row hove the lower bone quality

## Discussion

Using MDCT for clinical purposes in the present study, we evaluated BMD and four microstructural parameters of the greater tuberosity divided into six specific ROIs. The medial row showed higher BMD and BV/TV and lower SMI and trabecular separation than those of the lateral and far lateral rows. In these analyses, Pm showed the highest BMD and BV/TV and the lowest SMI and trabecular separation. These findings suggest that the medial row may have several advantages against anchor pullout compared with the lateral row. Pm, the posterior medial portion, possesses the most ideal plate-like structure with the highest bone quality. There are some biomechanical in vitro studies relating the bone quality to anchor pullout strength [12, 13, 18]. Besides, these findings are in accordance with previous reports of animal and cadaver models [18, 19]; Kirchhoff concluded that the portion with the highest bone quality was the posterior medial aspect of the greater tuberosity.

Surgeons have been conventionally limited in terms of where to place suture anchors because the site of tendon reattachment is influenced by the type and size of the rotator cuff tear, the degree of tendon retraction, and the amount of tendon mobilization during the rotator cuff surgery [7]. Furthermore, surgeons sometimes have an inevitable complication of failure as a result of anchor pullout. Djurasovic et al. [9] showed failure occurred in 10% of 80 cases as a result of anchor loosening or migrating. Kaar et al. [11] reviewed 8 failed shoulder repair cases and found 2 suture anchors originally implanted in the humeral head to be free-floating, which led to severe articular damage. Therefore, in case of older individuals with osteoporosis, it is important to consider the types and placement of anchors or selection of repair methods (the suture anchor technique versus the bone tunnel technique) in terms of bone quality. Recommendations in the literature regarding the optimum region for anchor placement in rotator cuff repair are controversial. Some articles have recommended placing suture anchors lateral and distal to the greater tuberosity because the bone stock is better in this area [7, 24]. In contrast, other studies have shown that a position medial to the tip of the tuberosity is the optimum region for anchor placement [6, 25]. According to the present study, the ideal region for anchor placement in terms of bone quality is the posterior medial portion of the greater tuberosity. We believe that the lateral row (Al, Pl) and far lateral row (Af, Pf) are unfavorable for suture anchor insertion, especially in patients with osteoporosis. Because of the bone quality characteristics of these patients, additional options for rotator cuff repair might be considered, such as the bone tunnel technique.

The limitation of this study should be noted. First, we don't have a healthy control group in order to compare bone quality. Our series had mainly small and 1 medium supraspinatus tear. As Kirchhoff et al. described, normally the anterior portion of the great tuberosity is weaker than the posterior one and the same happens for the medial part. Moreover, this may worsens with rotator cuff tear considering from the previous reports that the laceration of the tendon insertion leads to osteoporosis of the bone. Our results may reflect these facts, and to be more explicit about our results, we have to evaluate a healthy control group in further study. Lastly, our series had a small number of cases with retrospective review. However, our method has profound significance in terms of allowing for in vivo evaluation of the osteoporotic bone microstructure of each patient. Based on these data, our further investigation of measuring absolute value by using imaging phantom may help to determine the most appropriate operative procedure preoperatively and to decrease the rate of retears and reoperations that occur secondary to anchor pullout. In addition, this work may extend to other skeletal regions in which anchor fixation is required in trabecular bone. Understanding the individual and regional variance in bone quality among patients is of utmost importance, and the details of clinical applications are being assessed in our hospital for creation of personalized surgical protocols.

## Conclusion

We have reported in vivo evaluation of the bone microstructure of the greater tuberosity of the humeral head in patients with rotator cuff tears. According to the present study, the posterior medial portion possesses the most ideal plate-like structure with the highest bone quality, therefore the ideal region for anchor placement in terms of bone quality is the posterior medial portion of the greater tuberosity. Conversely, the lateral row and far lateral row of the tuberosity are the most critical area regarding tendon fixation in terms of stability due to the lower bone quality.

## Authors’ original submitted files for images

Below are the links to the authors’ original submitted files for images. Authors’ original file for figure 1 Authors’ original file for figure 2 Authors’ original file for figure 3 Authors’ original file for figure 4 Authors’ original file for figure 5 Authors’ original file for figure 6 Authors’ original file for figure 7 Authors’ original file for figure 8

## Abbreviations

BMD: Bone mineral density
MDCT: Multidetector row computed tomography
CT: Computed tomography
MRI: Magnetic resonance imaging
ROI: Regions of interest
BV/TV: Bone volume/total volume
SMI: Structure model index.
