# Supplementary figures and images for: In vivo microstructural analysis of the humeral greater tuberosity in patients with rotator cuff tears using multidetector row computed tomography
Source: BMC Musculoskelet Disord. 2014 Oct 21;15:351. doi: 10.1186/1471-2474-15-351 (PMC4216346; doi:10.1186/1471-2474-15-351)

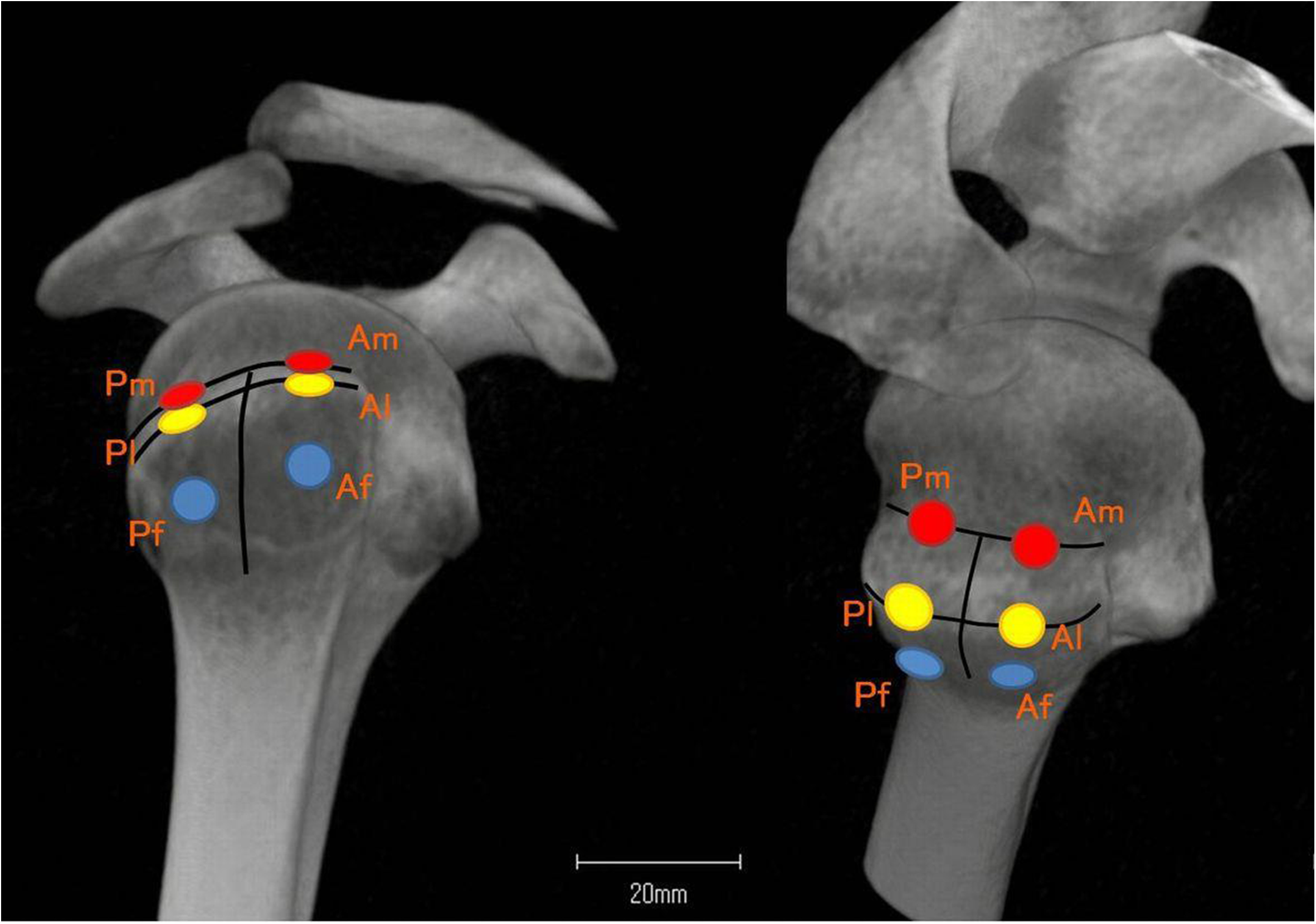

Supplement: Supplementary file 1 — Authors’ original file for figure 1 [file 12891_2014_2288_MOESM1_ESM.tif]

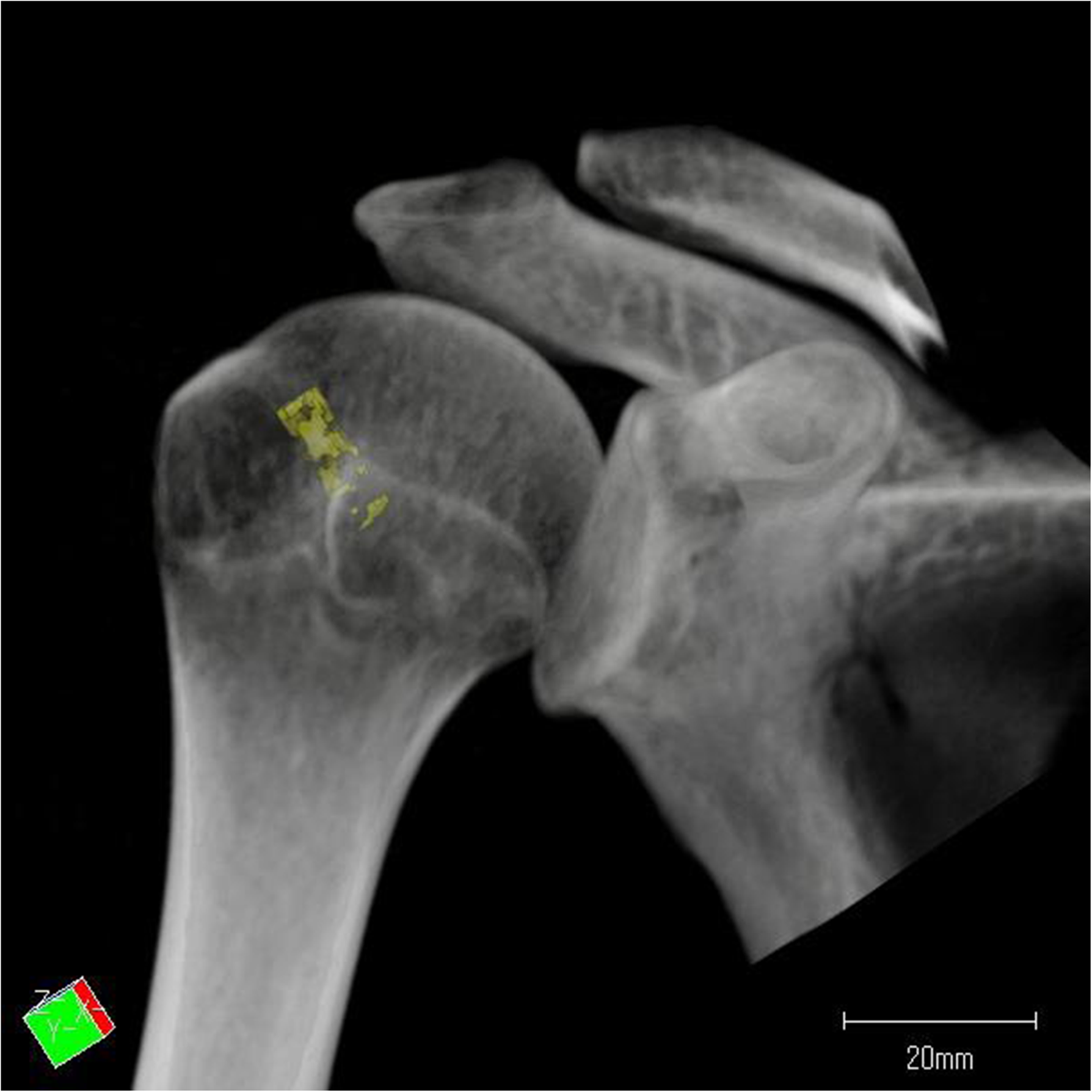

Supplement: Supplementary file 2 — Authors’ original file for figure 2 [file 12891_2014_2288_MOESM2_ESM.tif]

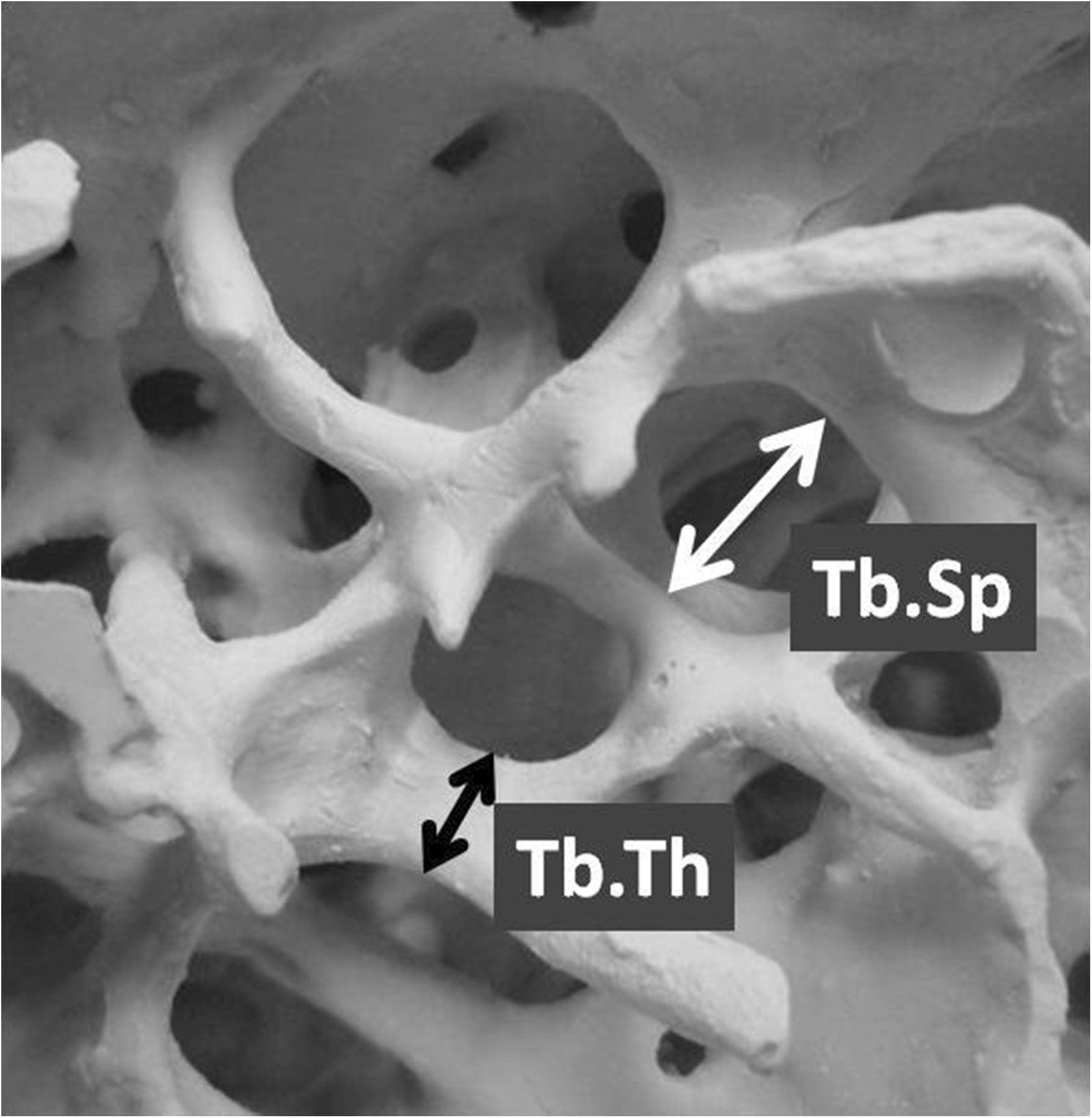

Supplement: Supplementary file 3 — Authors’ original file for figure 3 [file 12891_2014_2288_MOESM3_ESM.tif]

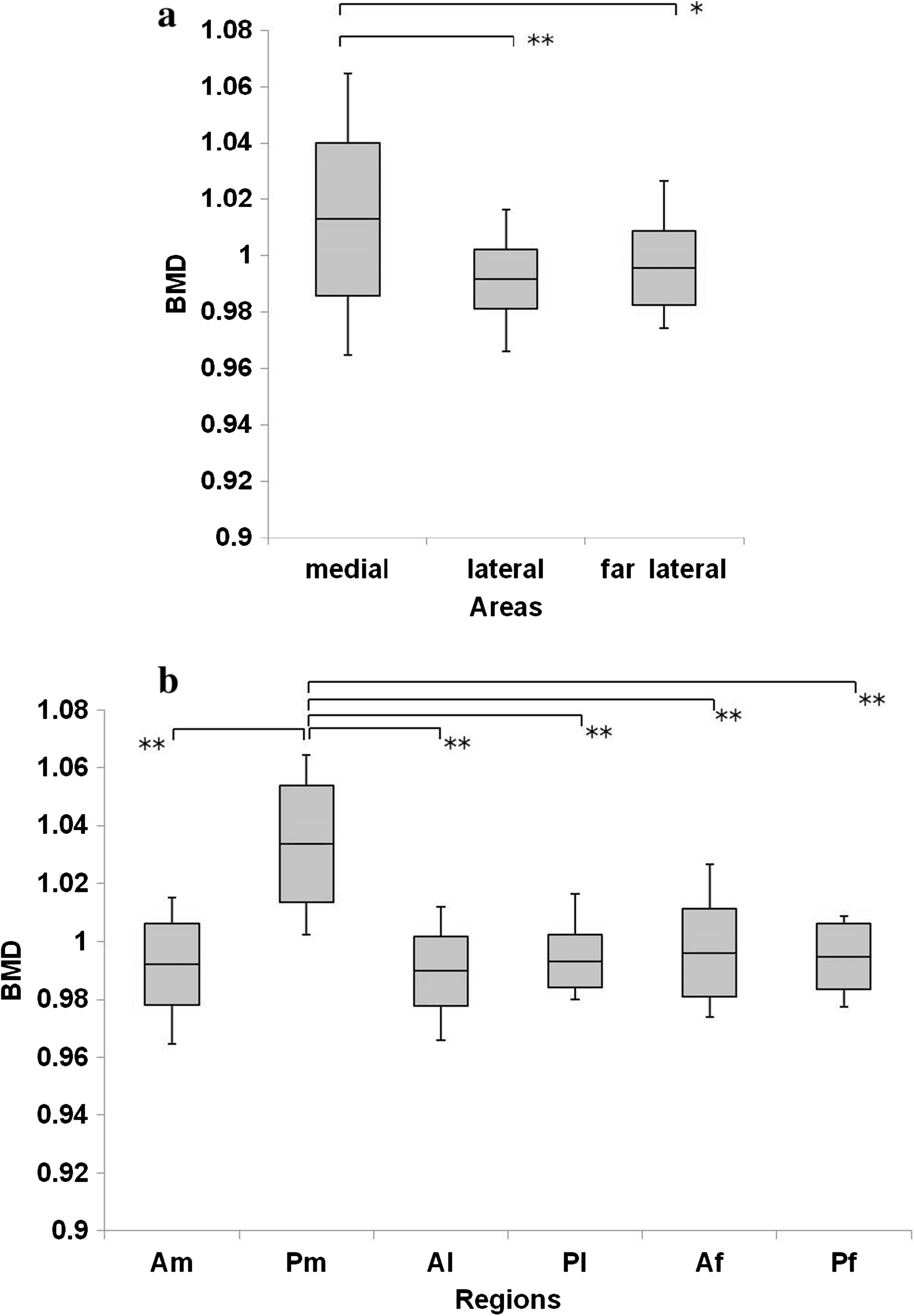

Supplement: Supplementary file 4 — Authors’ original file for figure 4 [file 12891_2014_2288_MOESM4_ESM.tif]

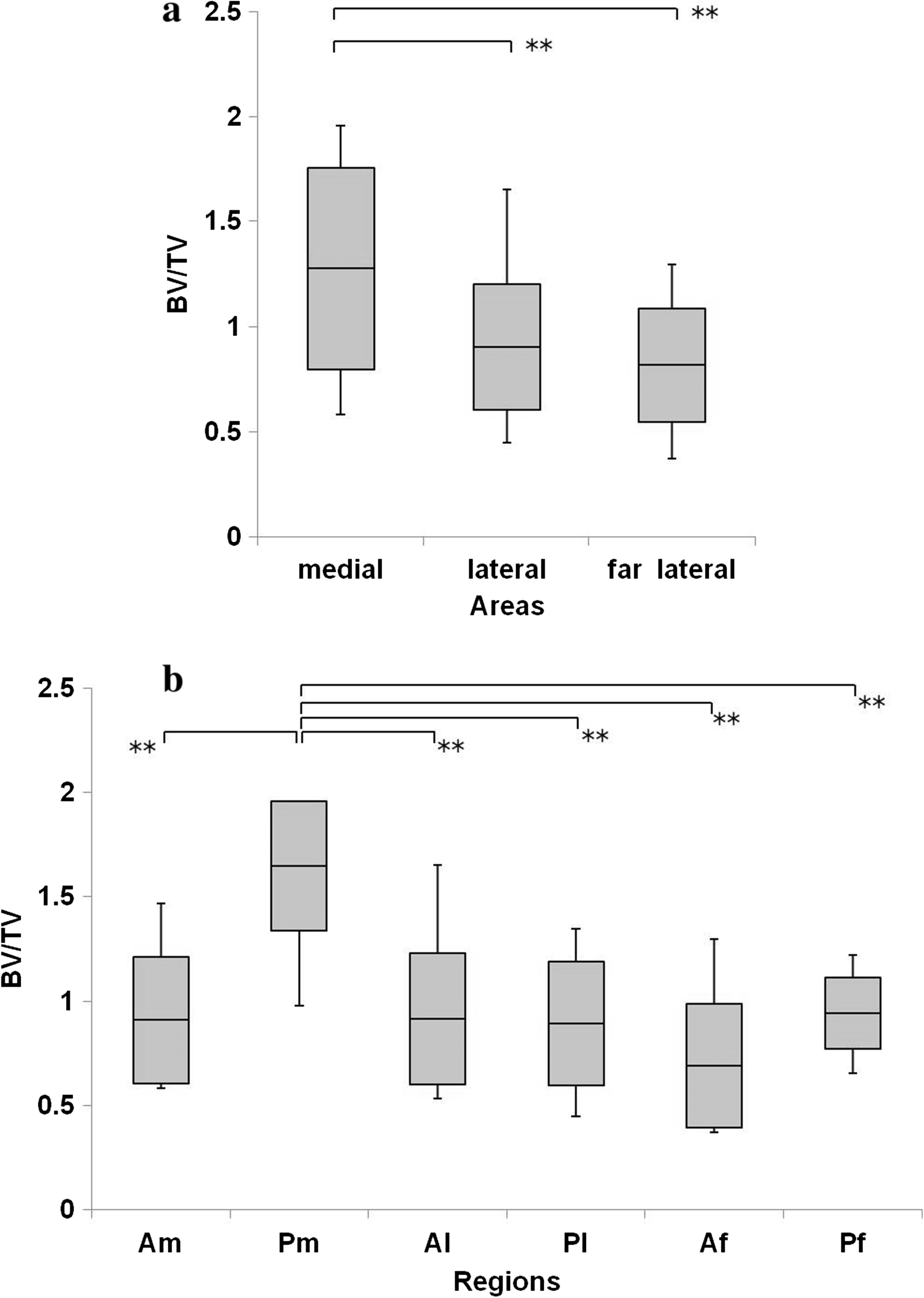

Supplement: Supplementary file 5 — Authors’ original file for figure 5 [file 12891_2014_2288_MOESM5_ESM.tif]

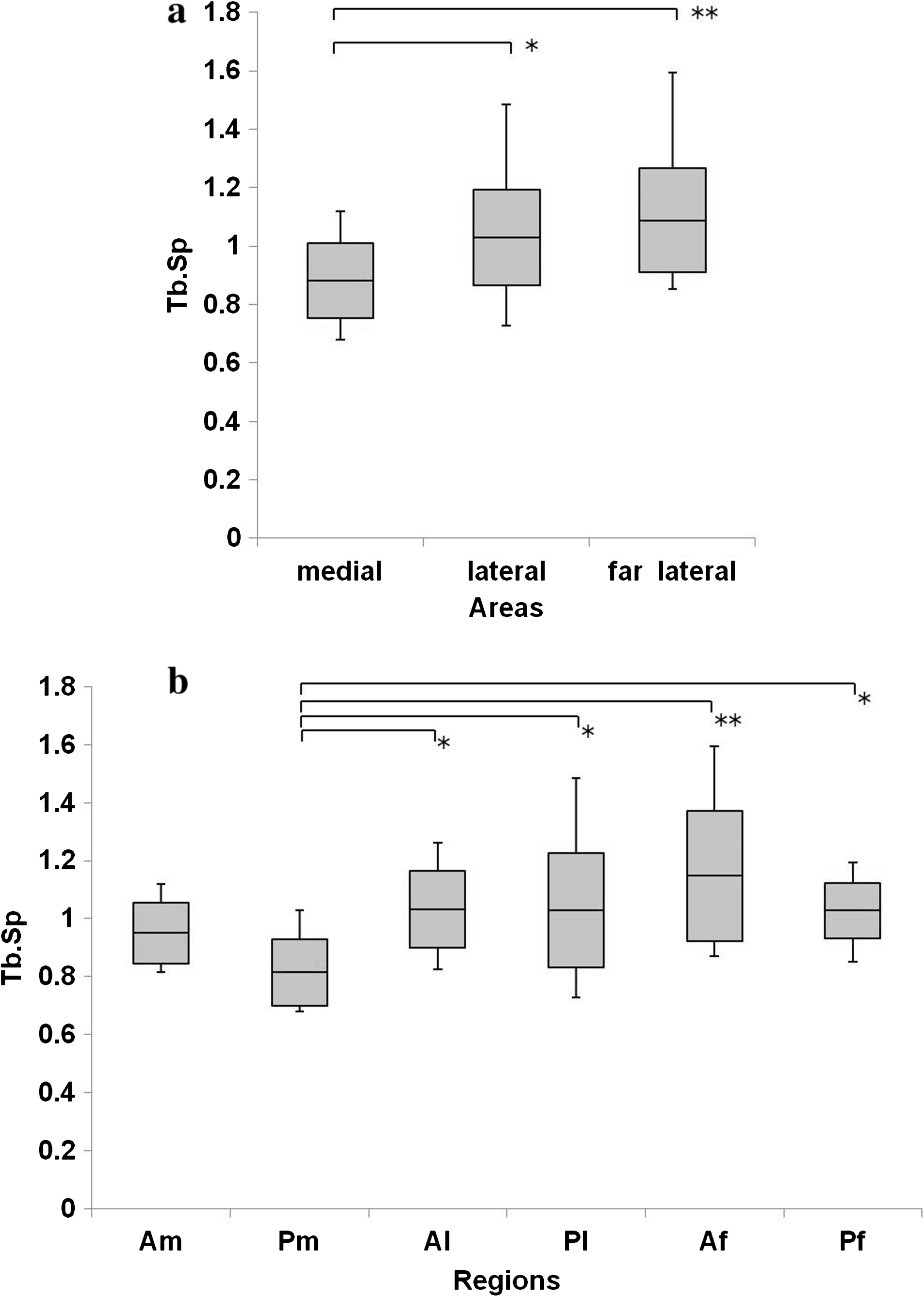

Supplement: Supplementary file 6 — Authors’ original file for figure 6 [file 12891_2014_2288_MOESM6_ESM.tif]

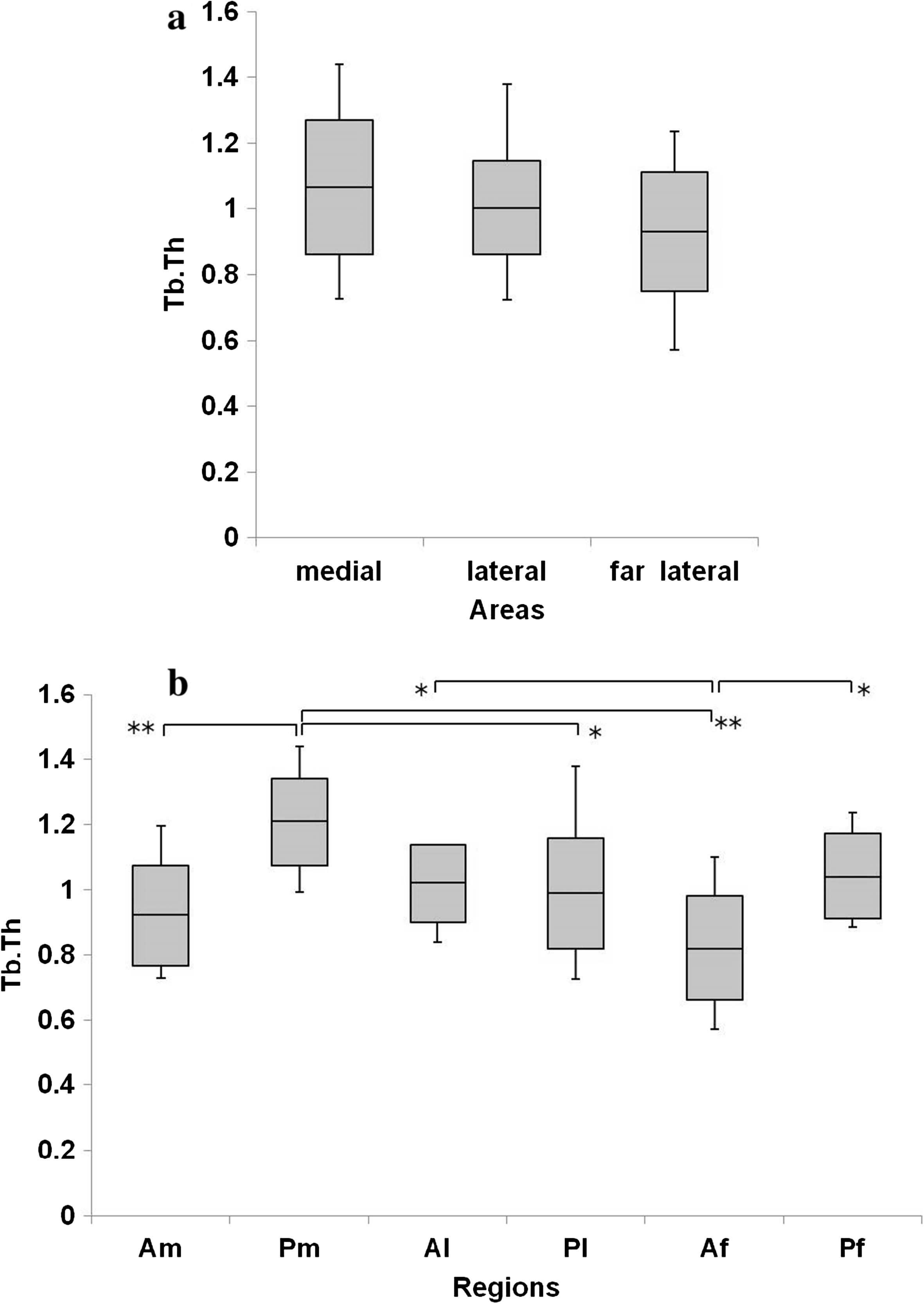

Supplement: Supplementary file 7 — Authors’ original file for figure 7 [file 12891_2014_2288_MOESM7_ESM.tif]

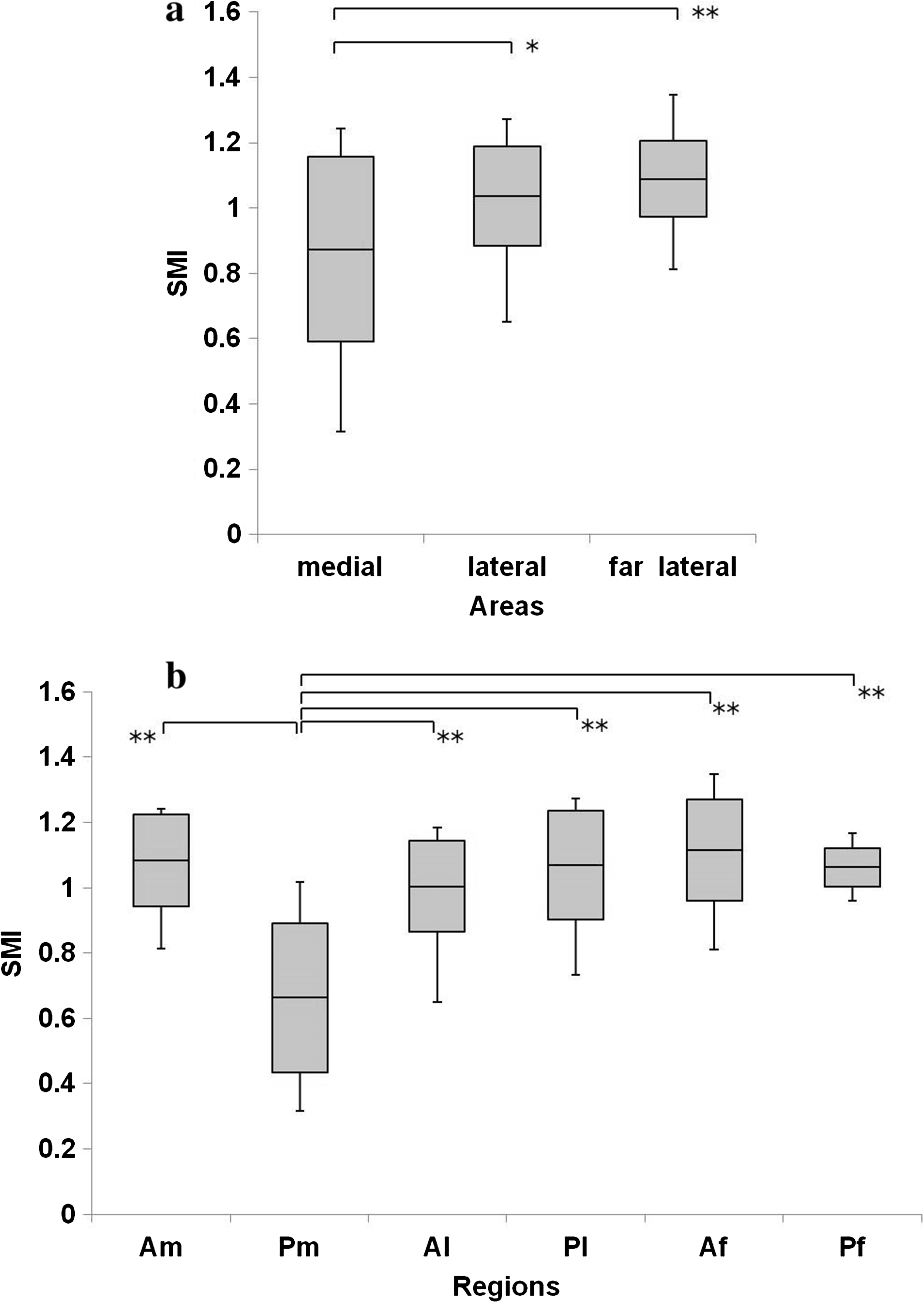

Supplement: Supplementary file 8 — Authors’ original file for figure 8 [file 12891_2014_2288_MOESM8_ESM.tif]
